# Cold adaptation mechanism in *Bactrocera dorsalis* (Diptera: Tephritidae) by regulating MAPK signaling and metabolic pathways

**DOI:** 10.1093/jisesa/ieaf109

**Published:** 2026-01-19

**Authors:** Ghulam Murtaza, Farman Ullah, Zihua Zhao, Zhihong Li

**Affiliations:** State Key Laboratory of Agricultural and Forestry Biosecurity, MARA Key Lab of Surveillance and Management for Plant Quarantine Pests, College of Plant Protection, China Agricultural University, Beijing 100193, China; State Key Laboratory for Managing Biotic and Chemical Threats to the Quality and Safety of Agro-products, Institute of Plant Protection and Microbiology, Zhejiang Academy of Agricultural Sciences, Hangzhou, P. R. China; State Key Laboratory of Agricultural and Forestry Biosecurity, MARA Key Lab of Surveillance and Management for Plant Quarantine Pests, College of Plant Protection, China Agricultural University, Beijing 100193, China; State Key Laboratory of Agricultural and Forestry Biosecurity, MARA Key Lab of Surveillance and Management for Plant Quarantine Pests, College of Plant Protection, China Agricultural University, Beijing 100193, China

**Keywords:** climate change, cold treatment, transcriptome analysis, DEG, RNAi

## Abstract

*Bactrocera dorsalis* (Hendel, 1912), a major invasive pest, survives under extreme climates through molecular and tissue-specific cold stress adaptations. In this study, we investigated the tissue-specific impacts of cold stress on the survival and molecular response of *B. dorsalis*. Results showed that cold stress had a significant effect on survival rates. The Kyoto Encyclopedia of Genes and Genomes pathway analysis indicated that signaling and metabolic pathways were activated by cold stress in the head and fat body during a transcriptome analysis. Under cold stress, 184 and 365 genes were differentially expressed in the head and fat body, respectively. RNA interference (RNAi)-mediated knockdown of transposon *Ty3-I Gag-Pol polyprotein* (*Ty3-I*) and *Ty3-G Gag-Pol polyprotein* (*Ty3-G*) in the head and fat body, significantly reduced the larval survival. Relative expression analysis revealed that expression of the *Ty3-I* and *Ty3-G Gag-Pol polyprotein* was greatly reduced in the head of cold treated larvae relative to controls (*dsGFP*) and that the expression level of *Ty3-I Gag-Pol polyprotein* in the fat body was not significantly reduced by cold stress. These results highlight the tissue-specific response of *Ty3-I* and *Ty3-G Gag-Pol polyproteins* in mediating cold stress responses and aid in understanding their importance in survival and stress adaptation. Additionally, the identification of important stress-responsive genes provides a foundation for the development of RNAi-based strategies for pest control using the targeted disruption of stress adaptation gene pathways for more effective control of *B. dorsalis* populations.

## Introduction

Increasing thermal stress due to global temperature fluctuations and extreme climate events pose increasing challenges for adaptation by ectothermic organisms such as insects. The survival of these organisms in a changing environment depends on their ability to withstand cold stress (John et al. 2024, Ullah et al. 2024). *Bactrocera dorsalis* (oriental fruit fly) is a major pest and is highly thermally adaptable (Ullah et al. 2022, Murtaza et al. 2025b), as a result of several ways to maintain cellular homeostasis under thermal stress conditions (Teets and Denlinger 2013, Yu et al. 2022, Zhao et al. 2024, Murtaza et al. 2025a). These adaptive responses involve heat shock proteins which help denatured protein fold and protect cells from thermal stress (Dou et al. 2017, Anantanawat et al. 2024). Other mechanisms such as autophagy, changing metabolism, or the presence of transposable elements (TEs) also make a large contribution to the insect’s ability to withstand thermal conditions (Signor et al. 2022). TEs can act as powerful sources of genetic and epigenetic variation, providing the flexibility needed for rapid adaptation to environmental stressors.

Genetic variability and rapid adaptation to environmental stressors are driven by TEs, including *Ty3-I* and *Ty3-G*, as well as characterized Ty3/Gypsy retrotransposons. These elements can introduce genetic variation to new genomic locations to alter gene function and regulation, which may facilitate adaptation to thermal extremes (Casacuberta and González 2013, Villanueva-Cañas et al. 2019). Insertion of TEs into various genomic regions generates mutations that increase genetic diversity, which confers adaptive advantage through response to changes in the local climate (Schrader and Schmitz 2019). More recent evidence indicates that TEs are not passive junk components of the genome, as previously thought, but active regulators of the genome, particularly during periods of stress. TEs in *Drosophila* species have even been shown to undergo neutral and positive selection as adaptive TE insertions (Rech et al. 2019) and *Drosophila melanogaster* increased TE activity during oxidative and radiation stress shows that these TEs are active and responsive to stress (de Oliveira et al. 2021) and probably work as responsive elements of a TE stress response system and network. Active elements also respond to the cellular stress response system. Other studies stress regulators and transcription factors reveal the enriched TE families further establish the flexible and dominant control and potential functions of TEs in alternate (flexible) stress/challenge-induced gene regulation (Villanueva-Cañas et al. 2019). Stress regulating gene expression across temperate and tropical *Drosophila* populations shows stress expression and TEs are geographically and environmentally correlated (Merenciano et al. 2025).

In *B. dorsalis*, evidence from both the genome and transcriptome indicates that retrotransposons *Ty3-I* and *Ty3-G* are some of the most actively transcribed *Ty3/Gypsy* retrotransposons under environmental stress (Majid et al. 2025). The presence of Gag-Pol conserved polyprotein domains and stress-induced transcription patterns imply that these retrotransposons may aid in the functional adaptation of the *B. dorsalis* in cold environments, potentially through the modification of cold-adaptive MAPK pathways and metabolism. Moreover, *Ty3/Gypsy* elements are also known to mediate the molecular mechanisms of epigenetic regulation through DNA methylation and histone shifting, both of which play pivotal roles in the dynamic regulation of genes in response to the season (Villalba de la Pena et al. 2021, Bodelón et al. 2023). Furthermore, TEs were also associated with modulating cold tolerance and immunological responses demonstrated in *Drosophila montana*, where TEs are involved in adaptation to temperature fluctuations (Tahami et al. 2024). Collectively, these insights provide a strong rationale for hypothesizing that *Ty3-I* and *Ty3-G* contribute to cold tolerance and adaptive genomic plasticity in *B. dorsalis*.

Considering the recent TEs importance in thermal adaptation, this study aims to investigate whether *Ty3-I* and *Ty3-G Gag-Pol polyproteins* play a role in cold adaptation of *B. dorsalis*. In particular, to elucidate how these transposons contribute to genomic variability and cold stress tolerance, which will pare into the mechanisms by which *B. dorsalis* cope with cold temperatures at the molecular level. This research will identify key genes and molecular pathways mediating cold tolerance using tissue-specific transcriptome analysis in combination with RNA interference (RNAi) technology. The findings will help us to understand how these adaptive mechanisms are responding to thermal stress and how we can develop more effective pest management strategies in a changing climate.

## Materials and Methods

### Insect Housing and Exposure to Cold Stress

*Bactrocera dorsalis* was collected from Guangzhou City, Guangdong Province, in southern China. The flies were maintained in an incubator at 27 °C with 65% ± 5% humidity and a 14-h light/10-h dark photoperiod. Distilled water and artificial food consisting of a sugar/protein solution in a 3:1 ratio were provided. To assess the effects of cold stress, third-instar larvae from the F10 population were exposed to cold treatment (CT) at 5 °C for 24 h. Each treatment was performed in 4-ml centrifuge tubes filled with 3 g of liquid diet to support survival during thermal exposure. The centrifuge tube lids were perforated to ensure proper ventilation. Each treatment was replicated 5 times, with 40 larvae in each replication. After 24 h of exposure to the respective temperatures, the larvae were given 1 h of thermal recovery, after which mortality was recorded. Surviving larvae were then dissected in an aseptic environment to collect head and fat body tissues. These tissues were quickly frozen in liquid nitrogen to preserve RNA integrity for subsequent analyses. Data were analyzed using SPSS version 25.0, with one-way analysis of variance followed by Tukey HSD *t*-test was used to compare differences among multiple treatment groups. The experimental design and cold stress exposure protocol described here were previously published by Murtaza et al. 2025b.

### RNA Extraction and Library Preparation for Transcriptome Sequencing

After larvae dissection and tissues, samples were sent to BMK Company Ltd on dry ice for transcriptome analysis after temperature treatments. TRIzol Reagent (Life Technologies, United States) was used to extract RNA, which was measured in terms of its concentration by using a NanoDrop 2000 spectrophotometer (Thermo Fisher). The RNA Nano 6000 Assay Kit (Agilent Technologies, United States) was used to assess RNA quality using the Agilent Bioanalyzer 2100.

For the RNA sequencing, 1 μg of total RNA from each sample was obtained using the NEBNext Ultra RNA Library Prep Kit for Illumina (NEB, United States) based on the manufacturer’s protocol for isolation of mRNA, clearing with polyT oligo: dT beads and cDNA synthesis with random hexamer primer and M-MuLV Reverse Transcriptase. DNA Polymerase I and RNase H were used to synthesize second strand cDNA from the first strand cDNA, which was diluted and adenylated, ligated to NEBNext adaptors, and enriched with remaining fragments of 250 to 300 bp using the AMPure XP system (Beckman Coulter, United States). The polymerase chain reaction (PCR) was carried out using the Phusion High First DNA polymerase for amplification. The libraries were purified again and their quality checked with Agilent Bioanalyzer. Clustering was performed using cBot Cluster Generation System of Illumina, and sequencing was carried out on the Illumina HiSeq platform with 125/150 bp paired-end reads.

### Transcriptome Analysis

Raw reads of fastq format (raw data) were run through house perl scripts and then used for clustering. After that, filtered the reads with adaptors, poly-N bases, and low-quality reads and got clean data (clean reads). At the same time and across all the clean data, we also computed Q20, Q30, and GC-content of the clean data, after which we used these computed values for all the analysis. With reference to NCBI Assembly #ASM78921v2, HISAT2 v2.0.4 was used to map clean paired-end reads to the whole genome sequence of *B. dorsalis*. Since splice junctions can be produced from HISAT2 by providing a gene model annotation file, HISAT2 was more appropriate for use here than other nonsplice mapping tools. Using HTSeq version 0. 9, we counted the read number that maps to each gene. The expression level of each gene was investigated, and expression of each gene was quantified by the fragments per kilobase of transcript per million mapped reads (FPKM) value of the gene according to its gene length and number of reads aligned to the gene. Therefore, the most widely used method to determine the gene expression level is FPKM, which is the read counts for the sequencing depth and the gene length in a sample simultaneously (Trapnell et al. 2009, Florea et al. 2013).

### Comparison of Differentially Expressed Genes and Gene Ontology

Normalized read counts were then performed using edgeR before differential gene expression analysis. DEGSeq R package (1.20.0) was used to identify differentially expressed genes (DEGs) with *P* values corrected by Benjamini–Hochberg method. Genes with a *P*-value < 0.05, false discovery rate < 0.005, and log2(Fold Change) > 1, were considered DEGs. GOseq was used for Gene Ontology (GO) enrichment analysis, and genes were considered to be significantly enriched if the corrected *P*-value < 0.05.

Kyoto Encyclopedia of Genes and Genomes (KEGG) was then used to test for statistical enrichment of DEGs for pathway analysis, and KOBAS software (Mao et al. 2005) was used for pathway analysis. Both known and novel transcripts were identified and assembled using Cufflinks v2.1.1 with transcripts being annotated. RNA-Seq data assembly was performed using the Reference Annotation based Transcript assembly method based on TopHat alignment.

### RNA Extraction, cDNA Synthesis, and Real-Time PCR

To validate transcriptome analysis findings, we measured the relative expression of the candidate gene in the head and fat body of *B. dorsalis*. mRNA was extracted from treated larvae using the RNAsimpleTotal RNA Kit (TianGen, China), and RNA concentration was determined using a NanoVue UV–V is spectrophotometer. Reverse transcription was performed with the PrimeScript RT reagent Kit (Takara, China), and cDNA was stored at −20 °C.

Real-time PCR was conducted using an ABI QuantStudio 6 Flex (Applied Biosystems) and SYBR Premix Ex Taq II (Takara, Japan). The *18s* gene served as the reference. Primers were designed using Primer Premier 6 (Table 1). The PCR reaction mix contained SYBR Green, forward and reverse primers and ddH2O. PCR conditions included 95 °C for 30 s, 40 cycles of 95 °C for 5 s and 60 °C for 34 s. A total of five biological replicates were used for statistical analysis, and the 2^−ΔΔCT^ method was applied. Data were analyzed using SPSS R25.0.0, with *t*-tests for group comparisons. Figures were generated using GraphPad Prism 8.0.

**Table 1.** Primer sequences used for RT-qPCR and dsRNA synthesis

Gene name	RT-qPCR primers
**Primers for head genes**	
***Ty3-G Gag-F***	ATTGTGGGTGTAACGCTGTTG
***Ty3-G Gag-R***	GTCGAATTACACTCAGATCAGGAA
***Ty3-I- Gag-F***	TGGAACAGCATACACCTCGT
***Ty3-I Gag-R***	CGGGATCATGGACTTCGGTA
***CIC IX2-F***	ACCCCAAGTCGCCGTTAAAA
***CIC IX2-R***	TGGAACGGCACTTACTTATCCA
***RFC1-F***	CAGTGCTCACCTGTTTCGTT
***RFC1-R***	GTGAACGCAACTTCCACACT
**Primers for fat body genes**	
***Ty3-G Gag-F***	ATTGTGGGTGTAACGCTGTTG
***Ty3-G Gag-R***	GTCGAATTACACTCAGATCAGGAA
***Ty3-I- Gag-F***	TGGAACAGCATACACCTCGT
***Ty3-I Gag-R***	CGGGATCATGGACTTCGGTA
***SH2B-F***	GTTCAGAGGCAGCACGTATG
***SH2B-R***	AAATTCACCTTTGCGCGTCT
***HRAS-F***	TGCGAACTGGAGAGGGATTT
***HRAS-R***	GCCGTCAACCAGTCAGAATT
**Gene name**	**dsRNA Primers**
***Ty3-G Gag-F***	TTCGGCTACTGTCTTCGCTTCCT
***Ty3-G Gag-R***	CTCCTCACCGCATCAGAATCAATCA
***Ty3-I Gag-F***	ATTGGACTCCTCACCGCATCAGA
***Ty3-I Gag-R***	GGCTACTGTCTTCGCTTCTTGGTT
***T7_Ty3-G Gag-F***	TAATACGACTCACTATAGGTTCGGCTACTGTCTTCGCTTCCT
***T7_Ty3-G Gag-R***	TAATACGACTCACTATAGGCTCCTCACCGCATCAGAATCAATCA
***T7_Ty3-I Gag-F***	TAATACGACTCACTATAGGATTGGACTCCTCACCGCATCAGA
***T7_Ty3-I Gag-R***	TAATACGACTCACTATAGGGGCTACTGTCTTCGCTTCTTGGTT

### *dsRNA* Synthesis and RNAi-Mediated Knockdown of *Ty3-I* and *Ty3-G*

For gene silencing, the *dsRNA* of ds*Ty3-I* and ds*Ty3-G* were utilized to suppress the target genes in *B. dorsalis* tissues, and *dsGFP* was used as a control. The *dsRNA* was synthesized using the T7 RiboMAX Express RNAi system from Promega with specific primers, including *Ty3-I*-*dsRNA*-F/R and *Ty3-I*-*dsRNA*-F/R-T7, *Ty3-G*-*dsRNA*-F/R and *Ty3-G*-*dsRNA*-F/RT7 (Table 1). Early third instar, 5-day-old larvae were fed 3 g of an artificial diet containing 30 µl of *dsRNA* solution (1,000 ng/µl) in a 4-ml tube. The lid of the tube had small holes that allowed air to flow through for the cold response study. Each treatment was repeated 5 times, and each replication contained 40 larvae. The larvae were allowed to feed on the artificial diet with incorporated ds*Ty3-I*, ds*Ty3-G*, and *dsGFP* for 96 h.

Generally, 20 larvae of *B. dorsalis* were dissected after 96 h of *dsRNA* feeding to collect head and fat body samples to quantify gene expressions. To understand the role of genes in cold hardening, the larvae were treated with cold hardening (5 °C) and control (27 °C) for 24 h and then moved for 1 h to 27 °C. The survival rate was calculated as mentioned above. The survived larvae were dissected to get the head and fat body tissues for qPCR to confirm the relative expression level of the target genes (qPCR protocol as mentioned above). As previously mentioned, both treatments were repeated 5 times. Data were analyzed using the *t*-test and the statistical package SPSS R 25. 0. 0. 0, contrasting the genes’ expression levels under each treatment. The data were analyzed, and the figures were graphed in GraphPad prism 8.0 software.

## Results

### Impact of Cold Stress on Survival of *B. dorsalis*

Based on our previous findings (Murtaza et al. 2025b), the oriental fruit fly larval survival was significantly reduced under cold treatment compared with the control.

### Differential Gene Expression under Cold Stress

**Fig. 1.**
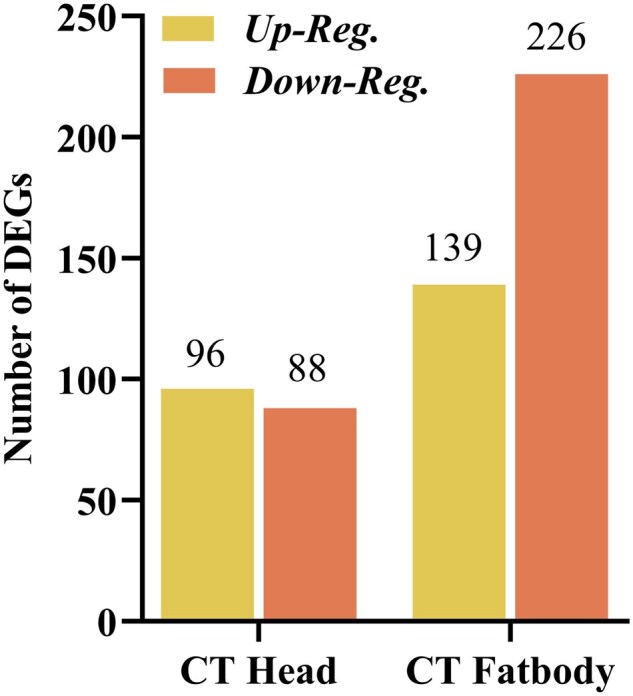
Differential gene expression under thermal stress.

Differentially expressed gene analysis indicates that thermal stress markedly altered gene regulation in the head and fat body tissues of *B. dorsalis* larvae. Results showed that there is a total of 184 (96 upregulated and 88 downregulated) DEGs after exposure to cold stress. While in the fat body, the number of DEGs was 365 (139 upregulated and 226 downregulated), as shown in Fig. 1.

### KEGG Pathway Enrichment and Transcriptomic Analysis Reveal Adaptive Responses to Cold Stress in *B. dorsalis* Larvae Heads and Fat Body

**Fig. 2.**
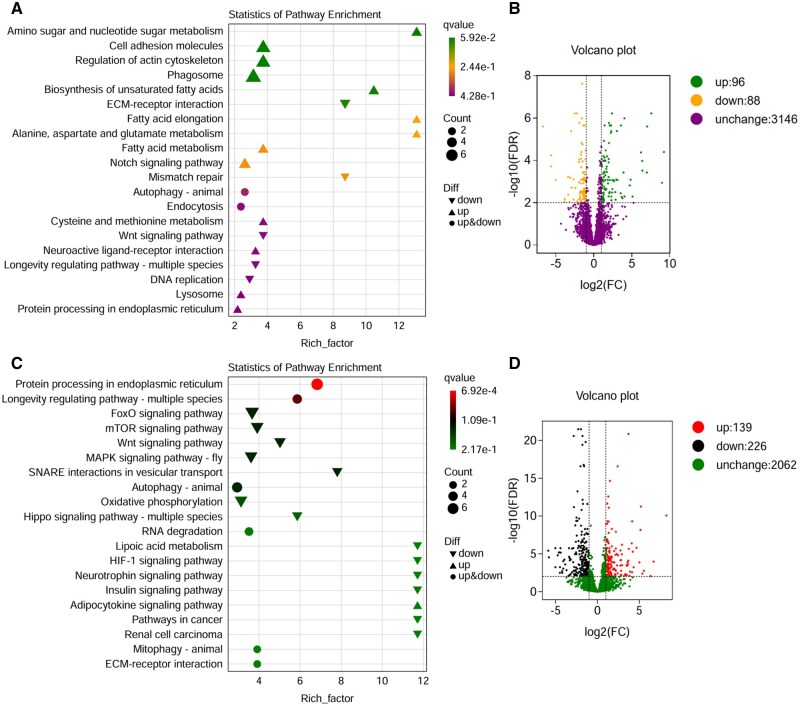
KEGG pathway enrichment and volcano plot of *B. dorsalis* larval heads and fat body under cold stress. A) The KEGG enrichment, B) the volcano plot in the larval head, C) the KEGG enrichment, and (D) the volcano plot in the larval fat body.

KEGG enrichment analysis and volcano plots give a global view of the transcriptional and pathway level response to cold stress in both larval heads and fat bodies. KEGG analysis in larval heads shows that many of these pathways are upregulated and enriched in terms of structural changes and immune responses (Fig. 2A). We find that these pathways have the highest gene contribution and statistical significance (low *q* values), indicating their importance in the cold stress response. Moreover, metabolic adaptations occur in the pathways of amino sugar metabolism, fatty acid metabolism, and in processes that are stress responses and recycling mechanisms, including in the pathways of mismatch repair, notch signaling and autophagy. Consistent with this, the volcano plot of larval head gene expression demonstrates major gene expression changes, with more upregulated genes with positive log2 (FC), −log10 (FDR) indicating pathway activation and downregulated genes with negative log2 (FC) indicating pathway suppression as showed in Fig. 2B.

KEGG enrichment analysis reveals several pathways related to stress response, including protein processing in the endoplasmic reticulum and the longevity regulating pathway, both of which have a high rich factor and statistical significance (low *q* values) in the larval fat body (Fig. 2C).

With little exception, several other key pathways, such as FoxO signaling pathway, autophagy and SNARE interactions in vesicular transport are also dramatically upregulated, which indicates their important contributions to cellular survival and vesicular transport in stress. A mixed response to stress and signaling pathways including MAPK signaling, mTOR signaling, Wnt signaling, and oxidative phosphorylation is observed with upregulated genes and downregulated genes. The volcano plot for fat body further shows a high level of gene expression change, with many upregulated genes and downregulated genes, as presented in Fig. 2D.

### GO Enrichment Analysis Highlights Adaptive Responses to Cold Stress in Larval Heads and Fat Body

**Fig. 3.**
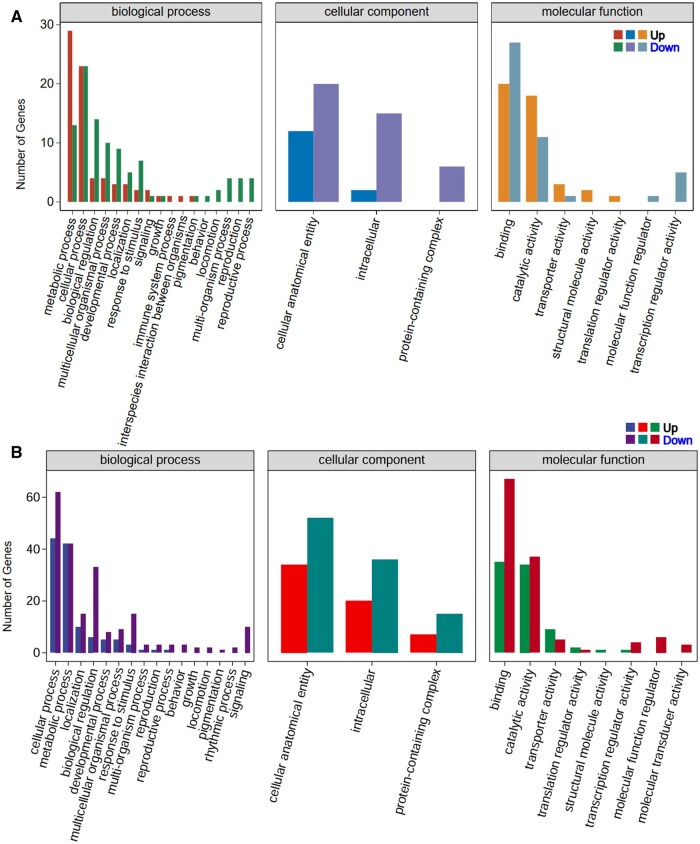
GO Enrichment analysis of larval heads and fat body under cold stress. A) Larval head: highlights metabolic processes, intracellular components, and molecular functions like binding and catalysis. B) Fat body: shows similar enrichments in metabolism, structural activity, and intracellular adaptations.

The GO enrichment analysis of larval heads and fat bodies under cold stress (Fig. 3) shows that transcriptional and functional adaptations were significant across biological processes, cellular components, and molecular functions. The biological process clustering of metabolic processes and cellular processes suggests active metabolic and regulatory responses in both tissues. They also show stress-related processes such as biological regulation and response to stimuli, which is due to tissue-specific adaptation to cold stress. Enrichment in intracellular regions and protein containing complexes identify intracellular activity and protein interactions necessary for stress adaptation as signals of enrichment in the cellular component category. The most enriched terms for molecular functions are binding and catalytic activity, showing the importance of molecular interaction and enzymatic activity under cold conditions (Fig. 3A and B). Furthermore, molecular transport and structural stability activities are exemplified by terms such as transporter activity and structural molecule activity. GO enrichment analysis overall reveals a tissue-specific and organized response to cold stress in that both larval head and fat body participate metabolically, structurally, and molecularly in their adaptation to cold stress.

### Validation of Transcriptome-Selected Genes Using qRT-PCR

**Fig. 4.**
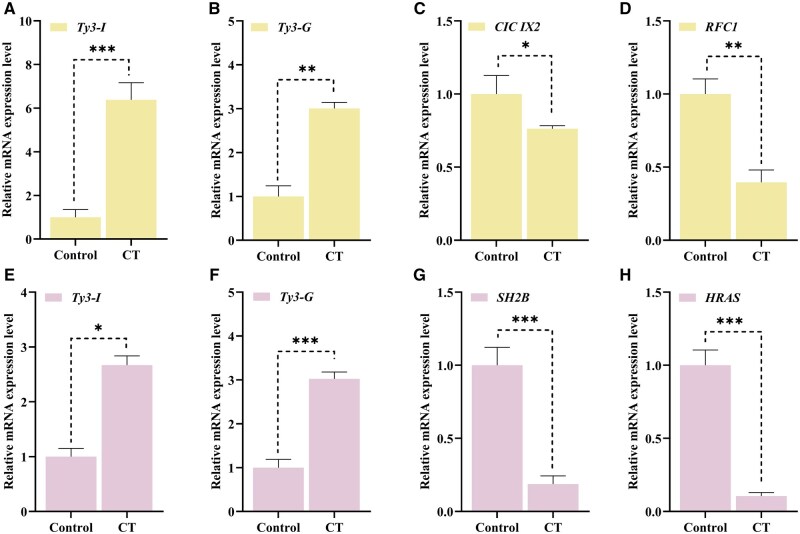
Relative mRNA expression level of the selected genes selected from transcriptome data. A–D) Relative expression level of *B. dorsalis* head gene under cold treatment conditions. E, F) Relative expression level of *B. dorsalis* fat body gene under cold treatment condition (*P < 0. 05, **P < 0. 01, ***P < 0.001).

The transcriptomic analysis revealed DEGs in *B. dorsalis* tissues under cold treatment, as presented in Fig. 4. Notably, Transposon *Ty3-I Gag-Pol polyprotein* (*Ty3-I*) and Transposon *Ty3-G Gag-Pol polyprotein* (*Ty3-G*) were upregulated in the head tissue during CT (Fig. 4A and B). This upregulation suggests potential roles in enhancing growth signaling and stress response mechanisms under cold stress conditions. Conversely, downregulation of the putative transcription factor *capicua isoform X2 (CIC IX2)* and *replication factor C subunit 1 (RFC1)* (Fig. 4C and D) under the same conditions may indicate a suppression of gene transcription regulation and nucleolar stress responses, respectively.

In the fat body tissue subjected to cold treatment, *Ty3-I* and *Ty3-G* also exhibited increased expression levels (Fig. 4E and F), potentially reflecting protective processes against cold-induced protein destabilization and activation of TEs as part of the stress response. Meanwhile, the downregulation of *SH2B adapter protein 1 (SH2B)* and *GTPase Hras (HRAS)* (Fig. 4G and H) suggests a reduction in intracellular sorting and signal transduction activities, which might be diminished under stress conditions at lower temperatures.

These findings align with the transcriptomic data and provide insights into the molecular mechanisms that enable *B. dorsalis* to adapt to thermal stress. The observed gene expression changes underline the physiological and evolutionary significance of thermal adaptation in this pest species, particularly in the context of fluctuating temperatures. Based on these results, *Ty3-I* and *Ty3-G* were identified as key genes involved in thermal adaptation and selected for further RNAi experiments targeting the head and fat body tissues.

### Effect of *Ty3-I* and *Ty3-G* Gene Silencing on the Survival of *B. dorsalis*

**Fig. 5.**
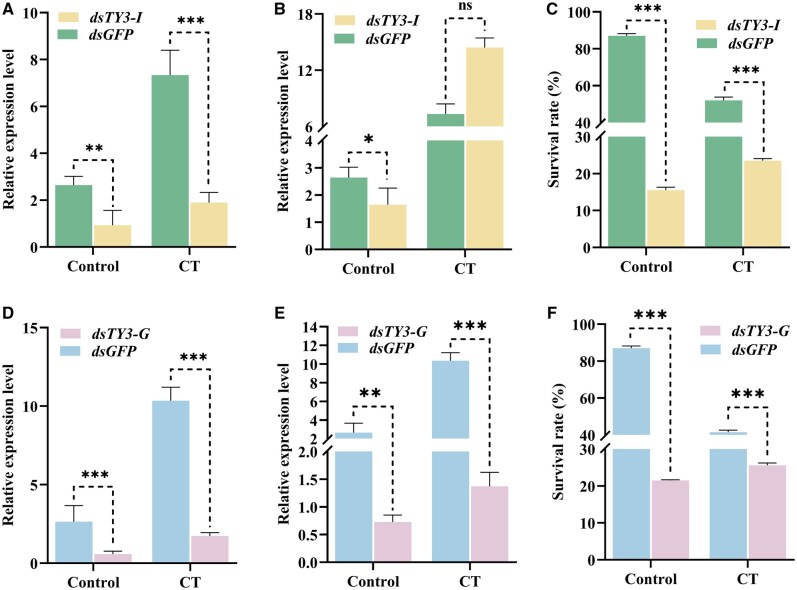
Relative gene expression analysis of *B. dorsalis* after RNAi. A, B) Relative expression levels of *dsTy3-I* after RNAi in the head and fat body under CT and control. C) Percentage survival of *B. dorsalis* after feeding on solution containing *dsRNA* of *GFP* and *dsTy3-I*. D, E) Relative expression levels of *dsTy3-G* after RNAi in the head and fat body under CT and control. F) Percentage survival of *B. dorsalis* after feeding on solution containing *dsRNA* of *GFP* and *dsTy3-G*. Data are expressed as mean ± SD (ns = nonsignificant, *P < 0. 05, **P < 0. 01, ***P < 0.001).

The RNAi-mediated knockdown of *Ty3-I Gag-Pol polyprotein* and *Ty3-G Gag-Pol polyprotein* revealed significant impacts on survival rates and gene expression profiles in specific tissues under cold stress conditions, highlighting their roles in stress adaptation as shown in Fig. 5. In particular, *Ty3-I* and *Ty3-G* knockdown in the head and fat body tissues showed pronounced effects during cold treatment. When insects were fed double-stranded RNA (*dsRNA*) targeting *Ty3-I* and *Ty3-G*, survival rates significantly decreased (80% and 78%) compared with *dsGFP* controls (Fig. 5C and F). A detailed tissue-specific analysis revealed that *dsTy3-I* expression was significantly downregulated in the head and fat body following RNAi treatment, as shown in Fig. 5A and B. Under CT, the expression levels of *dsTy3-I* were significantly reduced from 7.9-fold to 1.89-fold compared to the control group treated with *dsGFP* (Fig. 5A). However, in the fat body, expression levels were nonsignificantly upregulated under CT as shown in Fig. 5B. Similarly, for *dsTy3-G*, expression levels were markedly downregulated across all tissues after RNAi. Under cold stress, the expression of *Ty3-G* was consistently lower in all body parts compared to the *dsGFP* control, as shown in Fig. 5D and E.

## Discussion

**Fig. 6.**
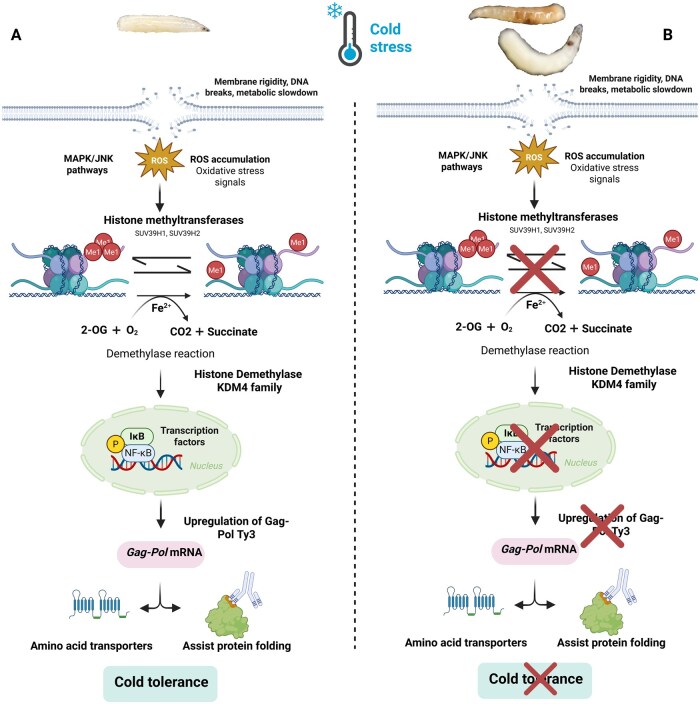
Functional mechanism of *Ty3-I* and *Ty3-G Gag-Pol polyprotein* in *B. dorsalis* after exposure to CT. A) Mechanism before RNAi. B) Mechanism after RNAi.

Cold acclimation in *B. dorsalis* is accompanied by large changes in both the transcriptome and metabolome, consistent with molecular and physiological increases in cold tolerance (Xie et al. 2023) as shown in Fig. 6. Cold stress differentially regulates pathways involved in the metabolisms of proline and glutathione, which are essential for stress resistance in fruit flies like *Drosophila* (MacMillan et al. 2016). Our transcriptome results demonstrated that metabolic shifts, such as the induction of cryoprotectants and changes in membrane fatty acids, are known to contribute to stabilizing cellular structures under cold stress in *B. dorsalis* (Ren et al. 2023). Additionally, the increased detection of trehalose during cold stress in other species, eg *Drosophila* is linked to increased cold tolerance (Yu et al. 2022). These results imply that metabolic changes are important components of cold adaptation in fruit flies like *B. dorsalis*. In addition, *B. dorsalis* larvae are resistant to cold stress, indicating the activation of particular molecular pathways, such as TEs such as *Ty3-I* and *Ty3-G* as shown in Fig. 6A. The insect’s ability to survive under harsh cold conditions may be due to these elements.

In addition to the metabolic adjustments, cold stress is probably associated with protective molecular responses as perceived in *B. dorsalis* similar as reported in *Drosophila*, the reversible cold hardening (RCH) response. Phosphorylation mediated signaling in key tissues, including the brain and fat bodies, is this process, to protect cells from apoptosis caused by cold (Yi et al. 2007, Teets and Denlinger 2016). Although the involvement of particular genes (such as the Frost genes) in the species of *Bactrocera* is yet unclear, thermal acclimation leading to cold tolerance by the pre-exposure to sub lethal temperatures is thought to be a main factor in the specie’s adaptation to cold stress (Ben-Yosef et al. 2023). Our results suggest that TEs, including *Ty3-I* and *Ty3-G*, were activated by cold stress to facilitate genomic plasticity and adaptation. This is consistent with findings in *D. melanogaster*, where TEs affect the transcriptional regulation of stress response genes to modulate the response of the organism to cold stress (Villanueva-Cañas et al. 2019). Incorporation of TE, eg *FBti0019985* in *Drosophila*, into promoter regions of stress-related genes, and modification of their activity can upregulate cold stress tolerance (Merenciano et al. 2016). TEs are either enhancers or regulatory elements to regulate host gene expression under environmental stresses like cold stress (Wheeler 2013). Furthermore, they allow for the rapid uptake of novel genetic material and erect stress-inducible regulatory networks to generate genetic variability that may be advantageous under cold stress (Capy et al. 2000, Casacuberta and González 2013). The ability to activate TEs under environmental stress (eg cold or heat shock) has been found to impact on gene regulation and may be advantageous relative to survival under harsh conditions. In species like *Drosophila suzukii*, cold acclimation induces a huge number of transcriptional changes and many genes are differentially expressed as a response to the cold stress (Enriquez and Colinet 2019). These transcriptional shifts are essential for insect survival, and TEs could facilitate such rapid adaptive processes by influencing gene expression of aspects of cold tolerance (Teets et al. 2012). Therefore, it is likely that TEs provide insects with the ability to cope with cold stress, allowing them to survive temperature fluctuations to survive cold environments and to be adaptable to cold environments.

The knockdown of *Ty3-G* and *Ty3-I Gag-Pol polyprotein* genes in insects may severely impair the retrotransposition process, leading to a loss of ability to mobilize stress-inducible transposons as shown in Fig. 6B. This disruption could inhibit the activation of essential gene networks responsible for cold stress responses, such as protein folding, membrane stability, and apoptosis suppression (Yi et al. 2007). However, during cold stress in insects such as *Drosophila*, vulnerable cells may also die through apoptosis, a process that is usually controlled by transposons, which activate to stabilize cellular structures and inhibit programmed cell death (Teets and Denlinger 2016). Thus, a decrease in transposon activity due to the knockdown of *Ty3-G* and *Ty3-I* would increase the likelihood of cold-induced apoptosis and lessen the ability of the insect to survive under cold stress (Fig. 6B).

Furthermore, *Ty3-G* and *Ty3-I* are also known to be essential for enhancing genomic placidity, promoting insects’ rapid response to environmental stresses. By reducing the insect’s success in adapting (Casacuberta and González 2013), the knockdown of these genes could limit the insect’s ability to create new genetic variability. RCH is one important adaptation to cold stress, relying on phosphorylation-mediated signaling to protect cells from damage caused by cold exposure (Teets and Denlinger 2016). Disruption of transposon activity may modulate this mechanism and, therefore, impair the expression of genes critical to cellular protection. We confirm this hypothesis by our results that both *Ty3-I* and *Ty3-G Gag-Pol polyproteins* are necessary for survival and adaptation to cold conditions and are localized in the head and fat body. Together, the reduced viability of *B. dorsalis* larvae after RNAi and the reduced expression of these genes in critical tissues highlight their essential role in establishing genomic stability, metabolic homeostasis, and stress response pathways. These results point to potential key regulatory roles for retrotransposon proteins in stress tolerance mechanisms and show their critical contribution to survival strategies against environmental challenges such as cold stress.

## Conclusions

Overall, our results suggested that these *Ty3-I* and *Ty3-G Gag-Pol polyproteins* are critical for larval survival and adaptation to cold stress and are localized in the head and fat body of the larvae. Collectively, these results demonstrated that these genes are crucial for regulating genomic stability, metabolic homeostasis, and stress response pathways in *B. dorsalis* as the decreased survival following RNAi treatment as well as decreased expression of these genes in critical tissues suggest. The observed effects also underscore the possible utility of retrotransposon proteins as key stress tolerance and survival strategy components under environmental challenge conditions, including cold stress. Future studies should investigate multiple temperature regimes and developmental stages to determine the precise molecular mechanisms by which these genes contribute to genomic stability and stress resilience. Additionally, exploring whether targeting retrotransposons such as *Ty3-I* and *Ty3-G* through RNAi could offer a novel strategy for pest management under cold environmental conditions would be valuable.

## Data Availability

All the data in this study which have been analyzed are provided within the context of the article and the supplementary material. Other supplementary data: for example, raw transcriptomic sequencing files which may be accessible on request from the corresponding author to support the findings of this study as well as to enhance the replicability of the research.
